# Cell Envelope Modifications Generating Resistance to Hop Beta Acids and Collateral Sensitivity to Cationic Antimicrobials in *Listeria monocytogenes*

**DOI:** 10.3390/microorganisms11082024

**Published:** 2023-08-07

**Authors:** Maarten Goedseels, Chris W. Michiels

**Affiliations:** Department of Microbial and Molecular Systems, KU Leuven, B-3000 Leuven, Belgium; goedseels.maarten@gmail.com

**Keywords:** *Listeria monocytogenes*, hop beta acids, natural antimicrobial, collateral sensitivity, cell envelope, MprF, dlt operon, antibiotic resistance, food preservative

## Abstract

Hop beta acids (HBAs) are characteristic compounds from the hop plant that are of interest for their strong antimicrobial activity. In this work, we report a resistance mechanism against HBA in the foodborne pathogen *Listeria monocytogenes*. Using an evolution experiment, we isolated two HBA-resistant mutants with mutations in the *mprF* gene, which codes for the Multiple Peptide Resistance Factor, an enzyme that confers resistance to cationic peptides and antibiotics in several Gram-positive bacteria by lysinylating membrane phospholipids. Besides the deletion of *mprF*, the deletion of *dltA*, which mediates the alanylation of teichoic acids, resulted in increased HBA resistance, suggesting that resistance may be caused by a reduction in positive charges on the cell surface. Additionally, we found that this resistance is maintained at low pH, indicating that the resistance mechanism is not solely based on electrostatic interactions of HBA with the cell surface. Finally, we showed that the HBA-resistant mutants display collateral sensitivity to the cationic antimicrobials polymyxin B and nisin, which may open perspectives for combining antimicrobials to prevent resistance development.

## 1. Introduction

*Listeria monocytogenes* is a Gram-positive foodborne pathogen that causes invasive listeriosis, an illness resulting in bacteremia, meningitis, meningoencephalitis, and stillbirth in pregnant women [1]. In the European Union (EU), invasive listeriosis is one of the most severe zoonoses monitored by EFSA and ECDC, with a case fatality of 13.7% and a hospitalization rate of 96.5% in 2021 [2]. Elderly, neonates, immunocompromised individuals, and pregnant women and their unborn children are especially susceptible to listeriosis [3,4]. While the disease is associated with high mortality, hospitalization, and morbidity, its notification rate in the EU was relatively low in 2021, at 0.49 per 100,000 population, a number similar to previous years [2]. This may be partly explained by the infective dose of *L. monocytogenes*, which is generally estimated to be medium to high for susceptible people and high for healthy individuals, although these numbers are difficult to establish in humans [5,6].

*L. monocytogenes* is remarkably tolerant to environmental stresses, being able to grow at temperatures ranging from −0.4 °C to 45 °C, a pH as low as 4.1, salt concentrations up to 13.9%, and in the presence of moderate levels of nitrite and other food preservatives [7,8,9]. The combination of this high environmental resilience with a versatile saprophytic lifestyle explains the ubiquitous occurrence of *L. monocytogenes* in water, soil, vegetation, and food processing environments [10,11,12,13]. Since *L. monocytogenes* is eliminated by heat treatment, risk products include in particular ready-to-eat (RTE) foods that can support the growth of the pathogen and that are not heated before consumption [2]. In these foods, the outgrowth of *L. monocytogenes* must be prevented by proper design, cold chain management, an appropriate shelf-life, and, when necessary, the use of food preservatives. However, like many other food additives, traditional food preservatives increasingly suffer from a negative image because they are believed—and sometimes proven—to have negative health effects. Well-studied examples of this are nitrates and nitrites, commonly used preservatives in meat products and cheeses, which have been linked to colorectal cancer [14]. This growing aversion to traditional preservatives has fueled the consumer demand for minimally treated food and stimulated research into alternative, more natural, and healthy food preservatives. In recent decades, researchers have explored various alternative food preservatives, and plant-derived compounds such as essential oils and their constituents from edible plants have received particular interest because of their natural origin [15,16,17]. However, while several of these compounds, such as linalool, vanillin, citral, thymol, and cinnamaldehyde, have been approved for use in foods as flavoring agents [18,19], their implementation as food preservatives often requires a separate approval procedure. Furthermore, their use is complicated due to factors such as limited solubility and stability, strong organoleptic properties, and/or low availability.

A promising plant-derived antimicrobial is hop beta acids (HBAs), a mixture of structurally related compounds (lupulone, adlupulone, and colupulone) extracted from the female cones of the hop (*Humulus lupulus*) plant. They received Generally Recognized As Safe (GRAS) status in the U.S. for use as an antimicrobial in certain cooked meat and poultry products, and they are also used to prevent microbial deterioration of thick juice, an intermediate product in the production of beet sugar [20,21]. Compared to other plant-derived antimicrobials, HBAs display an exceptionally strong antimicrobial effect against several Gram-positive bacteria in media and food products [22,23,24]. Therefore, despite their bitter flavor, the impact of HBA on the organoleptic properties of food is believed to be minimal when they are used to inhibit the growth of *L. monocytogenes* [25,26]. Additionally, HBAs are commercially produced in high volumes for a fraction of the cost of most traditional essential oils, and their compounds have a long history of use.

Despite the strong antimicrobial activity of HBA, the underlying mechanisms of their activity are not yet fully understood. Early research in *Bacillus subtilis* reported that hop constituents, including lupulone, induced primary membrane leakage and subsequent inhibition of several cellular functions, suggesting that the bacterial plasma membrane is the primary target [27]. Research on *Lactobacillus brevis*, a common beer spoilage bacterium, found that the activity of hop acids is strongly enhanced at low pH, indicating that the undissociated form is mainly responsible for the antibacterial action. Additionally, hop acids were found to act as ionophores that transport H^+^ across the bacterial plasma membrane, thereby altering ion gradients and lowering the intracellular pH [28,29,30]. The proton ionophore action was later confirmed using bilayer lipid membrane measurements [31]. Using a hop-adapted *L. brevis* strain, it was proposed that hop adaptation changed the metabolism to minimize ethanol stress, altered the plasma membrane composition and fluidity, and increased the incorporation of lipoteichoic acids in the cell wall, providing a reservoir of divalent cations [32]. Using proteomics, it was found that the stress response to hop in *L. brevis* includes a wide variety of intracellular changes, shifting the metabolism into an energy-saving mode, possibly due to divalent cation limitation of several cation-dependent enzymes [33]. Later, the same research group reported that hop compounds caused oxidative damage by inducing a manganese-binding-dependent transmembrane redox reaction. The authors proposed that iso-α-acids, the hop acids utilized in their study, pass the plasma membrane to form a complex with intracellular Mn^2+^, which subsequently transfers electrons through the plasma membrane to extracellular hop acids, and when it is reduced again, this leads to intracellular oxidative stress [34]. The authors suggested this generation of oxidative stress to be the primary antibacterial mode of action of iso-α-acids, while the ionophoric properties would be of minor importance [35].

While research on the antibacterial mechanism of hop acids exists, it mainly focuses on beer spoilage bacteria and iso-α-acids, which are more relevant in beer brewing. However, HBAs have stronger antibacterial activity and, therefore, offer more promise as potential food preservatives and therapeutic agents. The present research aimed to investigate the antimicrobial mode of action and the potential mechanisms of resistance development against HBA in the foodborne pathogen *L. monocytogenes* by conducting adaptive laboratory evolution (ALE). This revealed that the knock-out of the Multiple Peptide Resistance Factor (MprF) provides elevated tolerance to HBA. Interestingly, this protein is mainly known as a factor responsible for resistance towards cationic antimicrobial peptides (CAMPs) and antibiotics. Since the primary function of MprF is to mediate the L-lysinylation of phospholipids, this finding suggested that the activity of HBA against *L. monocytogenes* is dependent on the charge of the cell envelope, and this was further confirmed by demonstrating that loss of DltA, which is necessary for the alanylation of lipoteichoic acids, also results in increased HBA resistance.

## 2. Materials and Methods

### 2.1. Bacterial Strains and Growth Conditions

All bacterial strains used in this study are listed in Table 1. *L. monocytogenes* Scott A was obtained from the International Life Sciences Institute (ILSI) North America [36] and used as the wild-type (WT) reference strain and as the parental strain for the generation of mutants. *L. monocytogenes* strains were grown at 30 °C in brain heart infusion (BHI; Oxoid, Hampshire, UK). *E. coli* DH5α [37] and S17-1λpir [38] were used as cloning hosts and as donor strains for conjugational transfer of plasmid DNA to *L. monocytogenes*, respectively, and were grown at 37 °C in Luria–Bertani medium (LB; 10 g/L tryptone (Thermo Fisher Scientific, Waltham, MA, USA), 5 g/L yeast extract (Thermo Fisher Scientific), 5 g/L NaCl). The growth media were supplemented with 50 µg/mL kanamycin (Km; AppliChem GmbH, Darmstadt, Germany), 100 µg/mL ampicillin (Amp; Thermo Fisher Scientific), 5–50 µg/mL polymyxin B sulfate (PMB; AppliChem GmbH), or 10 µg/mL erythromycin (Ery; Acros Organics, Geel, Belgium) as required.

### 2.2. Construction of in-Frame Deletion Mutants and Ectopic Overexpression Mutants

In-frame deletion mutants were created in an *L. monocytogenes* Scott A genetic background, using the pHoss1 plasmid, which was first modified by integrating an RP4 origin of transfer (oriT) from pIMK2, enabling its conjugative transfer. Then, approximately 1 kb of the up- and downstream regions of the genes of interest were amplified using PCR with the A and B, and C and D primers of the respective genes (Table 2) and subsequently combined with linearized (primers pHoss1_F and R) pHoss1-oriT plasmid using Gibson assembly, producing a vector that carries the deletion cassette. The constructed pHoss1*Δgene* plasmids were subsequently conjugated from *E. coli* S17-1λpir to *L. monocytogenes*, after which the protocol described by Abdelhamed et al. was followed, leading to markerless in-frame deletions [40]. PCR amplification and Sanger sequencing were used to confirm the correct genotypes.

To create ectopic overexpression mutants of the genes of interest, a pIMK2 shuttle vector was employed [39]. The wild-type genes were amplified using PCR (primers gene_F and gene_R, or mprF_SalI and mprF_BamHI for *mprF*) and assembled in a linearized (primers pIMK_F and pIMK_R, or restriction enzymes SalI and BamHI for *mprF*) pIMK2 vector using Gibson assembly, or restriction and ligation for *mprF*. After verification with Sanger sequencing, *E. coli* S17-1λpir was used as a conjugative donor for plasmid transfer to *L. monocytogenes*. Correct genomic integration of the vector was confirmed by PCR using a primer at the integration site (NC16(2)) and a forward primer on the gene of interest. Additionally, to account for any potential impact of pIMK2 vector integration, strains with an integrated empty pIMK2 vector were created and used as controls.

### 2.3. Hop Beta Acids (HBAs)

Beta-rich hop extract 40% (Hopsteiner, Mainburg, Germany) was used as a source of HBA. It is a clear brown liquid containing 40.0% hop beta acids in 20% propylene glycol, and was stored in the dark at 4 °C. Fresh stock solutions of 0.1% HBA in ethanol were made weekly.

### 2.4. ALE Experiment to Isolate Mutants with Increased HBA Resistance

**Figure 1:**
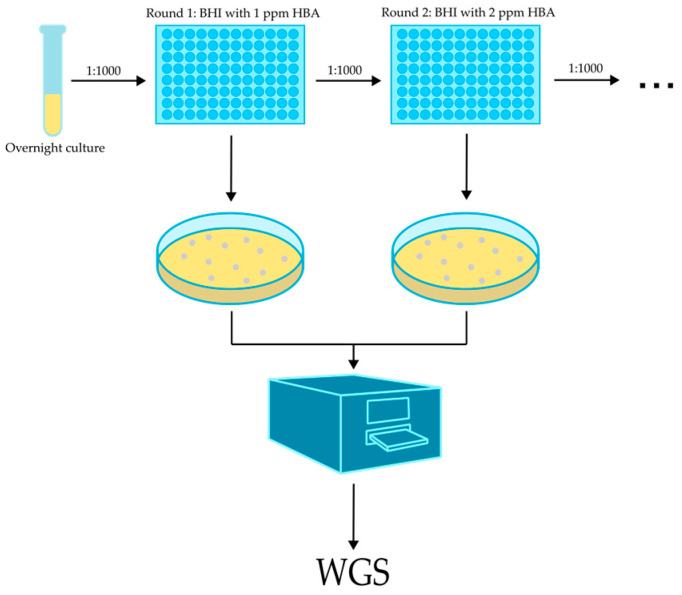
Schematic overview of adaptive laboratory evolution (ALE) experiment to isolate HBA-resistant mutants.

To isolate mutants with increased HBA resistance, an ALE experiment was conducted, as depicted in Figure 1. First, a colony of the WT *L. monocytogenes* Scott A strain was inoculated in 4 mL of BHI broth and grown overnight at 30 °C with shaking at 250 rpm. The culture was diluted 1000-fold in BHI containing 1 ppm of HBA and divided into 24 wells of a 96-well microplate. The cultures were incubated at 30 °C with shaking (250 rpm) until stationary phase (as monitored by OD620nm measurement) and subsequently diluted and regrown again for three more cycles, but alternating between 2 ppm and 1 ppm of HBA. As a control, some cultures were cycled in BHI without HBA. The addition of an equivalent amount of ethanol to these controls was omitted because prior analysis showed that these low concentrations of ethanol did not have any detectable effect on growth. As the experiment progressed, some of the 24 parallel cultures required a shorter time to reach the stationary phase, and these were plated on BHI agar to isolate individual colonies. The HBA resistance of several individual colonies was assessed in a growth assay in a microplate reader, and putative mutants with confirmed increased resistance were selected for whole genome sequencing (WGS).

### 2.5. Identification of Mutations

Genomic DNA (gDNA) was isolated for WGS using a Qiagen genomic DNA extraction kit (Qiagen, Hilden, Germany). Subsequently, the quantity and quality of the gDNA were checked using spectrophotometry (mySPEC, VWR, Haasrode, Belgium; Qubit, Thermo Fisher Scientific). Then, the gDNA was processed using an Illumina DNA Prep kit (Illumina, San Diego, CA, USA) and a Nextera DNA CD Index kit (Nextera, Juno Beach, FL, USA), and the prepared libraries were run on an Illumina MiniSeq (Illumina, 150 bp paired-end reads). CLC Genomics Workbench 12.0 (Qiagen) was used for quality checking, trimming, assembly of the reads to the reference genome, and identification of mutations. Identified mutations were confirmed using PCR amplification and Sanger sequencing.

### 2.6. Growth and MIC Assays

Growth curves were established using an automated microplate spectrophotometer (Multiskan Ascent or Multiskan FC, Thermo Fisher Scientific). Overnight stationary phase cultures were adjusted to equal turbidity (OD600nm) using an Ultrospec 10 Cell Density Meter (Biochrom, Cambourne, UK) and diluted 1000-fold (unless otherwise mentioned) in fresh BHI with or without (control) HBA. Aliquots of 200 µL were transferred to a 96-well microplate, and the plate was sealed using a transparent gas-impermeable foil. The microplates containing the cultures were incubated in a photometric reader at 30 °C, and the OD620nm or OD630nm (device-dependent) was measured every 10 or 15 min, after shaking. The Excel add-in DmFit 3.5 (Quadram Institute Bioscience, Norwich, UK), based on the model of Baranyi and Roberts [41], was used to determine the growth parameters of each culture, including the maximum growth rate (µmax), lag phase duration, and maximum OD (ODmax).

The minimum inhibitory concentration (MIC) of antimicrobials was defined as the lowest concentration where there was no detectable growth of any replicate after 24 h and was determined using the same protocol as for the growth assays. MIC assays were conducted for HBA (Hopsteiner), nisin (Sigma-Aldrich, Saint Louis, MO, USA), polymyxin B (AppliChem GmbH), sodium deoxycholate (Acros Organics), and sodium dodecyl sulfate (SDS; Applichem).

### 2.7. Survival Assay

The bactericidal activity of HBA was assessed by incubating three replicate 1000-fold diluted stationary cultures in potassium phosphate buffer (pH 7.0, 10 mM) with different concentrations of HBA, and counting survivors by plating on BHI medium after 3 h. Ethanol was added to the control treatment in an equivalent amount as for the highest HBA concentration used (64 ppm).

### 2.8. Statistical Analysis

Growth parameters of growth assays were calculated using the DMfit3.5 Excel add-in for at least three biological replicates. Using R Statistical Software (v4.2.0) [42], Shapiro–Wilk tests were used to test for normal distribution; afterward, the differences between means were established using ANOVA and post hoc Tukey’s HSD. Compact Letter Displays were generated using R packages multcomp (v1.4.23) [43] and multcompView (v0.1.8) [44]. For the survival assay, to examine the difference in survival between WT and mutants and between HBA concentrations, two-tailed *t* tests were performed using Excel. p values of <0.05 were considered statistically significant.

## 3. Results

### 3.1. HBA Has a Concentration-Dependent Growth-Inhibitory Effect on L. monocytogenes

**Figure 2:**
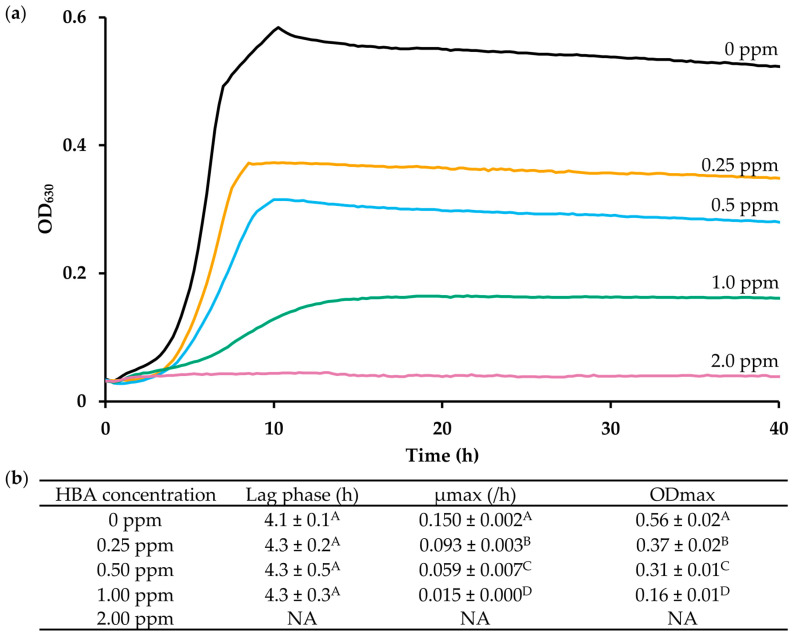
HBA has a concentration-dependent effect on growth of *L. monocytogenes*. (**a**) Growth curves of WT strain in BHI with increasing concentrations of HBA measured at 30 °C (n = 3). Only curves representing the mean of the replicate cultures is shown for clarity. (**b**) Lag phase duration, maximum growth rate (µmax), and maximum optical density (ODmax) of the growth curves shown in (**a**). Values followed by a common letter are not different at the 5% level of significance.

Figure 2 shows growth curves and growth parameters of *L. monocytogenes* WT Scott A in BHI at 30 °C with increasing HBA concentrations. The MIC is 2 ppm, and this is the lowest concentration at which there was no detectable growth after 24 h of incubation. At sub-MIC concentrations (from 0 to 1 ppm), there was a progressive, concentration-dependent decrease in the maximum growth rate (µmax) from 0.15 to 0.015, and in the maximum optical density of the cultures from 0.56 to 0.16. Under the conditions of this experiment, the lag phase duration was not affected at sub-MIC concentrations, unlike what is the case for many other plant-derived antimicrobials [45,46].

### 3.2. HBA Resistance Is Increased by Loss of Function Mutations in mprF

An ALE experiment was conducted to isolate mutants with increased HBA resistance. After two rounds of subculturing in the presence of sub-inhibitory concentrations of HBA (corresponding to approximately 20 generations), two mutants, HBA2.2 and HBA2.7, which exhibited increased growth in the presence of HBA, were isolated.

**Figure 3:**
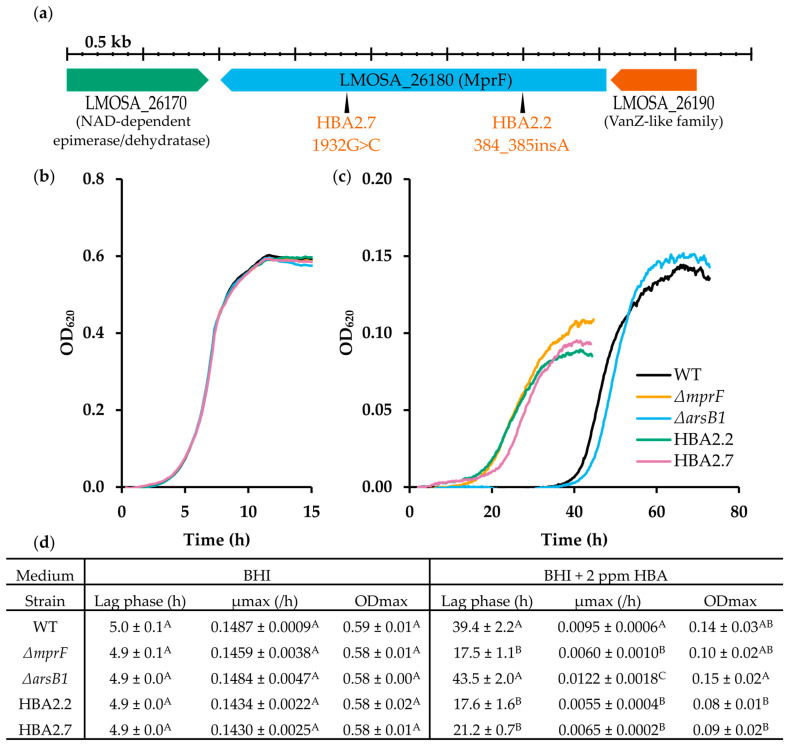
The evolved mutants, HBA2.2 and HBA2.7, and the constructed *mprF* deletion mutant display increased resistance to HBA. (**a**) The location of the *mprF* mutations of both evolved mutants is shown with black triangles. (**b**) Growth curves of WT, HBA2.2, HBA2.7, and the *mprF* and *arsB1* deletion mutants in BHI (*n* = 3). (**c**) Growth curves of the same strains in BHI supplemented with 2 ppm HBA at 30 °C (*n* = 3). Only curves representing the mean of the replicate cultures are shown for clarity. (**d**) Lag phase duration, maximum growth rate (µmax), and maximum optical density (ODmax) of the growth curves are shown in (**b**,**c**). Values followed by a common letter are not different at the 5% level of significance.

WGS of mutant HBA2.2 revealed a g.384_385insA mutation in *mprF* (NCBI Genbank sequence no.: CM001159.1; locus tag: LMOSA_26180), which results in a reading frame shift and the formation of an early stop codon, p.K129X (Figure 3). The *mprF* gene encodes a Multiple Peptide Resistance Factor, a bi-functional bacterial resistance protein responsible for lysinylation, and subsequent flipping of phospholipids over the membrane. It confers resistance to some antibiotics and cationic antimicrobial peptides (CAMPs) in several Gram-positive bacteria including *L. monocytogenes* [47]. While the exact structure of the *L. monocytogenes* MprF protein remains undetermined, the general topology with several transmembrane domains seems well preserved between species [48]. The premature stop codon caused by the p.K1269X mutation of HBA2.2 is predicted to be located in the N-terminal-membrane-embedded flippase domain and most likely to cause a loss of function. Additionally, a g.636_637delA in *arsB1* (LMOSA_2270) was also present in mutant HBA2.2, also causing a frameshift with an early stop codon (p.L225X). This gene is annotated as the arsenical-resistance protein (359 AA), a putative permease responsible for extruding arsenic from the cytoplasm [49]. Mutant HBA2.7 exhibited a g.1932G>C substitution in *mprF*, resulting in a p.E644D amino acid substitution, but no other changes. The p.E644D mutation is predicted to be located on the highly conserved cytosolic C-terminal synthetic domain, which catalyzes the aminoacylation of the phosphatidylglycerol [48,50,51]. The impact of this substitution on the functionality of the MprF protein is unclear.

To confirm the influence of the *mprF* and *arsB1* mutations on the phenotypes of HBA2.2 and HBA2.7, the deletion mutants of both genes were constructed from WT Scott A, hereinafter referred to as *ΔmprF* and *ΔasrB1* mutants. The growth curves and corresponding growth parameters of the evolved strains and both deletion mutants in BHI broth and BHI supplemented with 2 ppm HBA were established and compared to WT (Figure 3). In BHI, there was no discernible difference in the growth parameters between the HBA mutants, the deletion mutants, and the WT. However, in the presence of 2 ppm HBA, both HBA2.2 and HBA 2.7 were able to grow after a lag phase of 17.6 h and 21.2 h, respectively. For the WT, the present experiment, which had an extended duration of 3 days, confirmed complete growth inhibition by 2 ppm HBA for about 40 h (Figure 2) but additionally revealed the resumption of growth after this time (lag phase 39.4 h). Remarkably, the mutants’ maximum growth rates and ODmax remained lower than those of the WT. Both deletion mutants displayed normal growth in BHI broth but, in 2 ppm HBA *ΔmprF*, showed a reduced lag phase indistinguishable from HBA2.2, while *ΔarsB1* had a lag phase similar to WT. The *ΔmprF* mutant and HBA2.2 were slightly more tolerant than HBA2.7, possibly because the missense mutation in the latter may have caused only a partial loss of MprF activity. Altogether, the data consistently show that loss of MprF activity strongly reduces the lag phase duration in 2 ppm HBA, but slightly decreases µmax and ODmax. On the other hand, loss of ArsB1 had no effect on the lag phase but slightly changed the shape of the exponential growth phase and, possibly as a result of this, increased the calculated growth rate in the presence of HBA. Although this increase was significant in our experiment (*p* = 0.041), the difference with WT was small, and therefore, it was decided to not further investigate the role of ArsB1 in this work but to focus on MprF.

### 3.3. Loss of DltABCD Also Increases HBA Resistance

The ability of MprF to mediate resistance to cationic antimicrobial peptides and antibiotics has been at least partly attributed to the fact that it introduces positive charges on the cell surface by lysinylating phospholipids, thereby causing the repulsion of positively charged antimicrobials [47,52,53]. *L. monocytogenes* also has a *dltABCD* operon, which, together with *mprF*, is regulated by the two-component system VirRS [54]. DltABCD catalyzes the D-alanylation of teichoic acids and, thus, like MprF, also introduces positive charges on the cell surface [55]. Since HBAs carry a net negative charge at neutral or slightly acidic pH, we hypothesized that their antimicrobial activity may also be modulated by the cell surface charge, but in an opposite way as for cationic antimicrobials. If this is the case, loss of DltABCD would be predicted to increase the resistance of *L. monocytogenes* to HBA, similar to loss of MprF. To corroborate this hypothesis, *ΔdltA* and *ΔmprF*/*ΔdltA* mutants were constructed. DltA catalyzes the activation of the D-alanyl moiety and its binding to DltC and is, therefore, essential for D-alanylation of teichoic acids [56]. The *ΔmprF* and *ΔdltA* mutants were also genetically complemented by chromosomal integration of a pIMK2 vector containing the WT *mprF* or *dltABCD* genes. As a control, the empty pIMK2 vector was chromosomally integrated into the same location in the WT, *ΔmprF*, *ΔdltA*, and *ΔmprF*/*ΔdltA* (notation “/pI” after strain name).

**Figure 4:**
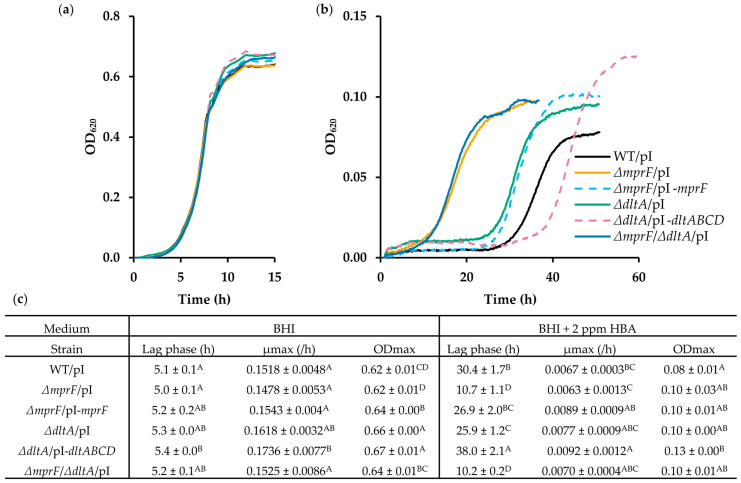
Loss of DltA also increases HBA resistance. (**a**) Growth curves of WT strain and *mprF* and *dltA* single and double deletion mutants with integrated pIMK2, and genetically complemented single deletion mutants in BHI at 30 °C (*n* = 3). (**b**) Growth curves of these strains in BHI supplemented with 2 ppm HBA (*n* = 3). Only curves representing the mean of the replicate cultures are shown for clarity. (**c**) Lag phase duration, maximum growth rates (µmax), and maximum optical densities (ODmax) of the growth curves shown in (**a**,**b**). Values followed by a common letter are not different at the 5% level of significance.

Figure 4 shows the growth of these strains in BHI broth without and with 2 ppm HBA. In BHI, all mutant strains grew equally well as WT/pI. In 2 ppm HBA, however, the deletion of *dltA* yielded an HBA-resistant phenotype reflected by a shorter lag phase as was the case for *ΔmprF*, but the level of resistance was lower. The reintroduction of the *dltABCD* operon in *ΔdltA* rendered the mutant sensitive again, with a lag phase time (38.0 ± 2.1 h) even exceeding that of WT/pI (30.4 ± 1.7 h). The reintroduction of *mprF* in the *ΔmprF* mutant also reversed the resistant phenotype of the mutant (lag time 26.9 ± 2.0 h). The double deletion mutant displayed the same level of resistance as the *ΔmprF* mutant.

**Figure 5:**
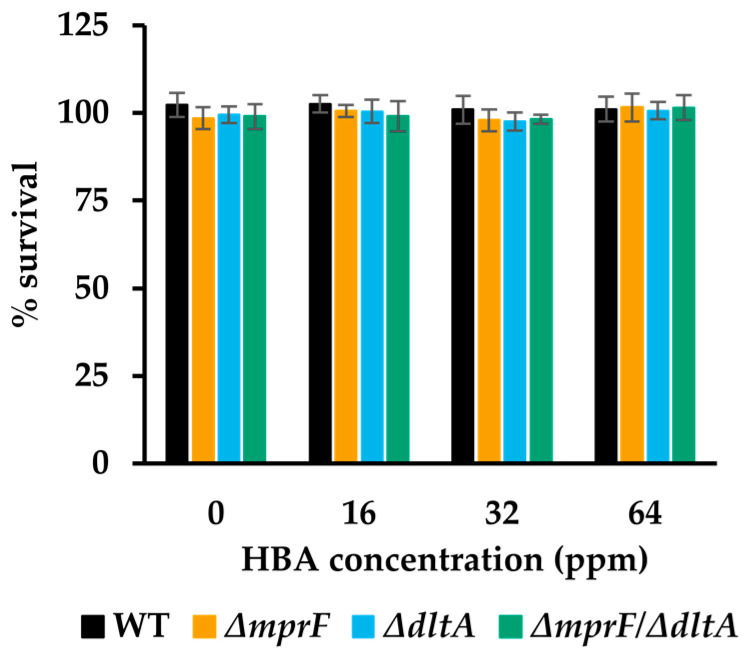
Survival of WT and deletion mutants after incubation in phosphate buffer with high concentrations of HBA over 3 h (*n* = 3). Error bars represent standard deviation.

A possible explanation for the long lag phase in the WT strain at 2 ppm HBA, and the ability of *mprF* mutants to reduce this lag phase, is that HBA has bactericidal activity and kills a fraction of the population in the first hours of exposure, after which the surviving cells can adapt and resume growth. To test for a possible bactericidal effect, we incubated stationary phase cells in phosphate buffer with high concentrations of HBA and enumerated the survivors after 3 h of incubation at 30 °C by plate counting. No bactericidal effect of HBA could be detected, even at 64 ppm, i.e., 32 times the MIC value of the WT strain (Figure 5).

### 3.4. HBA Resistance of ΔmprF/ΔdltA Is Maintained at Low pH

**Figure 6:**
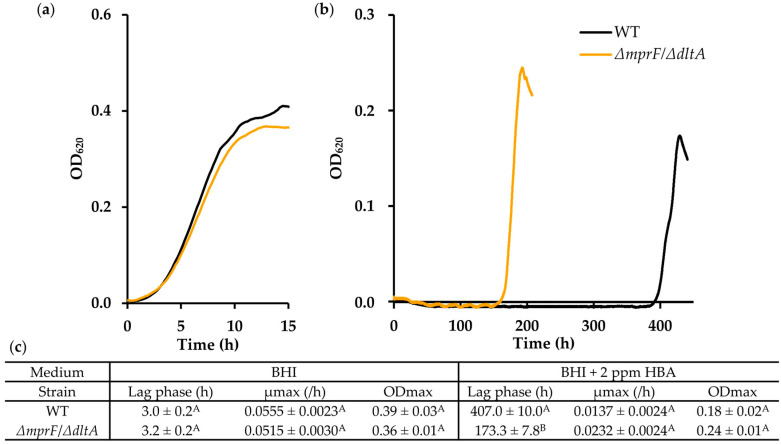
HBA resistance of the *mprF*/*dltA* double mutant is maintained at pH 5.4. (**a**) Growth curves of WT and *ΔmprF*/*ΔdltA* in BHI with a pH of 5.4 at 30 °C and (**b**) in BHI with 2 ppm HBA (*n* = 3). Only curves representing the mean of the replicate cultures are shown for clarity. (**c**) Lag phase duration, maximum growth rates (µmax) and maximum optical densities (ODmax) of the growth curves shown in (**a**). Values followed by a common letter are not different at the 5% level of significance.

The activity of HBA and other hop-derived antimicrobials is known to increase at low pH because a higher fraction of weak acids is undissociated under these conditions, which facilitates their passage through the plasma membrane [28]. Since we hypothesized that the HBA resistance in the MprF and DltA mutants may result from an increased electrostatic repulsion between HBA and the cell wall, it would follow that this resistance would diminish at a lower pH where most of the HBA and acidic cell surface groups are uncharged. To verify this prediction we compared the growth of WT and the *ΔmprF*/*ΔdltA* double mutant in BHI in the presence and absence of 2 ppm HBA at pH 5.4. Since the activity of HBA was strongly enhanced at this low pH, we had to increase the start inoculum of both strains tenfold compared to other growth experiments to obtain reproducible growth curves. As Figure 6 reveals, the double mutant appears to have a slight, though not significant, growth defect in BHI at pH 5.4 without the addition of HBA. As expected, the lag phase of the WT in the presence of 2 ppm HBA was much longer than in unacidified BHI (407.0 ± 10.0 h), but the mutant retained a high level of resistance, with a lag phase of less than half (173.3 ± 7.8 h).

### 3.5. HBA-Resistant Mutants Display Collateral Sensitivity to Some Cationic Antimicrobials

While MprF and DltABCD are mainly studied for their role in generating resistance against several cationic antimicrobials, our results indicate that their activity has an opposite effect on HBA resistance in *L. monocytogenes*. This is an interesting example of collateral sensitivity, where resistance to one antimicrobial is mechanistically linked to sensitivity to one or more other antimicrobials. In relation to MprF and DltABCD, and at least at neutral pH, this effect may be related to charge interactions between the antimicrobials and the cell surface. To determine whether this effect can be generalized, we performed MIC assays for some additional anionic and cationic antimicrobials (Table 3). While *ΔmprF*, *ΔdltA*, and *ΔmprF*/*ΔdltA *displayed lower MIC values for both tested cationic antimicrobials, polymyxin B and nisin, their MIC values for the anionic antimicrobials SDS and sodium deoxycholate were identical as for the WT strain. This indicates that the deletion of *mprF* or *dltA* does not induce a general resistance to anionic antimicrobials in *L. monocytogenes* and that the resistance mechanism to HBA might be more complex than solely charge-based repulsion.

## 4. Discussion

In this work, we investigated mechanisms of resistance against HBA in *L. monocytogenes* by evolving and characterizing HBA-resistant mutants and found that loss of function of the phosphatidylglycerol lysyltransferase MprF increased the resistance against HBA. Additionally, we demonstrated that also loss of the D-alanylation of lipoteichoic acids, mediated by DltABCD, increased the HBA resistance. While, in line with previous studies, both mutants showed increased sensitivity to the cationic antimicrobials nisin and polymyxin B, they did not exhibit increased resistance to other anionic compounds besides HBA, like SDS and sodium deoxycholate.

HBA is known to have strong antimicrobial activity against *L. monocytogenes*, and the MIC value of 2 ppm determined in our work in BHI at 30 °C is in line with previous reports. Kramer et al. studied a commercial HBA extract similar to the one used in our work and reported MIC values for *L. monocytogenes* DSM15675 of 12.5 ppm at pH 7.2 and 1.6 ppm at pH 5.0 after 18 h incubation at 37 °C in Mueller–Hinton broth [23]. Another study reported an MIC of 6.3 ppm for HBA in Trypticase Soy Broth–Yeast Extract against a cocktail of *L. monocytogenes* strains after 24 h at 37 °C [57]. Larson et al. tested the inhibitory effect of several hop extracts again *L. monocytogenes* Scott A and reported growth inhibition for 1 ppm of an extract containing about 30% colupulone, 65% lupulone, and adlupulone in BHI at 37 °C for 43 h [58]. The MIC values of HBA are in the same range for several other Gram-positive bacteria and are similar to that of many antibiotics rather than that of most plant-derived antimicrobials, which are typically in the mM range MIC [59,60,61].

Hop is a traditional ingredient of beer that contributes to a bitter flavor and microbiological stability. Nevertheless, some lactic acid bacteria (LAB) can spoil beer because they have evolved hop resistance, and one of the commonly implicated mechanisms involves multidrug transporters, such as HorA and HorC [62]. Behr, Gänzle, and Vogel succeeded in adapting an *L. brevis* strain to high concentrations of hop iso-α-acids. Throughout their evolution experiment, the culture’s resistance steadily increased over 60 days from 17.2 µM to 86 µM iso-α-acids. Interestingly, their hop-adapted MW 1.465A strain displayed a lag phase and growth rate that were nearly independent of the iso-α-acid concentration [32]. Our own evolution experiment yielded two independent *L. monocytogenes* HBA-resistant mutants already after two subcultures in the presence of HBA, corresponding to approximately 20 generations. To our knowledge, these are the first reported *L. monocytogenes* mutants with increased hop resistance.

Both evolved mutants had a different *mprF* mutation, and by constructing a deletion mutant, we were able to causally link the HBA resistance to MprF loss of function. The function of MprF has been mainly investigated in *Staphylococcus aureus* but has been confirmed in several other Gram-positive bacteria, including *L. monocytogenes*. In these bacteria, the protein mediates resistance to CAMPs and several cationic antibiotics, such as vancomycin, gentamicin, and moenomycin [53,63,64]. It is proposed to do so by lysinylating phospholipids and subsequently flipping them to the outer leaflet of the cytoplasmic membrane, thus increasing the positive charge of the membrane and causing the repulsion of cationic antimicrobials [47,50]. Additionally, MprF may play a broader, more complex role in *L. monocytogenes* virulence and physiology since its knock-out increased invasiveness in CaCo-2 intestinal cells, as well as biofilm formation on solid surfaces and motility at 37 °C [50,65].

*L. monocytogenes*, like many other Gram-positive bacteria, can also introduce positive charges on its surface by the D-alanylation of teichoic acids, which is mediated by the four proteins encoded by the *dltABCD* operon [55]. The knock-out mutants of *dltA* are sensitive to cationic antimicrobials, have severely impaired virulence, and display decreased adhesion to various cell lines [54,66]. Interestingly, *mprF* and the *dlt* operon are both regulated by the two-component system VirRS, a major regulator associated with *L. monocytogenes* cell invasion and virulence [54]. A study of the in vivo transcriptional response of *L. monocytogenes* in a mouse infection model showed 13 out of 17 VirR-regulated genes, including *mprF* and the *dlt* operon, to be upregulated, leading the authors to conclude that VirR is the second most important virulence regulator in *L. monocytogenes*, after PrfA [67]. Using a *dltA* deletion mutant, we demonstrated that the *dlt* operon has a comparable, but smaller, role in HBA resistance as *mprF*. Furthermore, *dlt* overexpression increased HBA sensitivity, further confirming the negative effect of these proteins on HBA tolerance. The D-alanylation levels of teichoic acids vary greatly between species and strains and are dependent on the growth conditions [68,69], so these effects of *dltA* deletion and *dlt* operon overexpression may be due to a low basal D-alanylation in the parental strain in our experimental conditions, resulting in only a small effect on cell envelope charge, but further research is required to confirm this hypothesis.

A possible explanation for the increased HBA resistance upon loss of MprF and/or DltABCD activity is that this renders the charge of the cell surface more negative, leading to increased repulsion of anionic antimicrobials like HBA. If this is correct, the effect of the mutations would be predicted to diminish at low pH, where the balance is again shifted towards more positive charges, both for HBA and on the cell surface. However, contrary to our expectation, the double mutant remained strongly resistant to HBA in comparison to the WT at pH 5.4. To further corroborate whether the observed MprF and DltABCD-based resistance was due to charge-based repulsion, the resistance to some additional cationic and anionic antimicrobials was determined. In accordance with the literature, the *mprF*, *dltA*, and *mprF*/*dltA* deletion mutants displayed increased sensitivity to the cationic antimicrobials nisin and polymyxin B [70,71]. However, their sensitivity to the anionic compounds SDS and sodium deoxycholate was unaltered compared to the WT. Similarly, in studies with LAB that are resistant to hop compounds, no cross-resistance against other antibacterial compounds, including weak acid food preservatives, solvents, antibiotics, and ionophores was detected [29,31]. In contrast to our findings, the deletion of *mprF2*, one of the two *mprF* orthologs of *Enterococcus faecalis* ([72]), was found to have increased survival when challenged with SDS, but surprisingly, this deletion mutant also did not differ from the parental strain in daptomycin resistance [73]. Together, this evidence suggests that the HBA resistance of our mutants may be partly due to electrostatic repulsion but probably involves additional effects of the mutations on the cellular physiology.

Although HBA and other hop extracts are generally considered to have a bacteriostatic effect, some researchers have reported a bactericidal effect against *L. monocytogenes* [24,57]. In our study, no inactivation of the WT and any of the mutant strains was observed upon a 3 h challenge with high concentrations of HBA (up to 64 ppm) in a buffer at pH 7. This eliminates the possibility that the observed differences in lag phase between the WT and the mutants (e.g., Figure 4) are due to the partial inactivation of the inoculum at the start of the experiment and confirms that the mutants have a genuinely increased HBA resistance.

Structurally related hop acids most likely share a similar antimicrobial mechanism because there is cross-resistance and sensitivity between them in bacteria [29]. It is, therefore, interesting to compare our findings for HBA with the proposed mode of action of iso-α-acids (isomerized humulone molecules), which were proposed to inhibit *L. brevis* in at least two ways. Firstly, they were shown to function as ionophores that transport H^+^ into the cell, leading to a dissipation of the transmembrane proton gradient and, especially in an acidic environment, to intracellular acidification [30,31,33]. Secondly, they may act as redox uncouplers by supporting a Mn^2+^-based transmembrane redox reaction. According to this mechanism, iso-α-acids that enter the cells through the membrane would form an intracellular complex with Mn^2+^ ions that has reducing properties and passes electrons via a membrane carrier to the hop acids at the outer side of the membrane, which act as electron acceptors at low pH. When the intracellular oxidized Mn-hop acid complex is subsequently re-reduced, this generates oxidative stress [34].

As teichoic acids contribute to general cation homeostasis by binding divalent cations [74,75,76], and the abrogation of the D-alanylation of these anionic polymers increases their ability to bind to divalent cations [77,78], the resistance mechanism of the *ΔdltA* mutant may rely on increased cation retention of the cell wall. Since one of the main proposed antibacterial activities of hop acids is divalent cation depletion caused by cation complexation, we hypothesize that loss of D-alanylation of teichoic acids reduces this effect by causing higher retention of cations in the cell envelope, thus increasing their availability and facilitating their re-uptake. This theory is further supported by the finding that a hop-resistant *L. brevis* strain displays increased lipoteichoic acid content of the cell wall, which increases divalent cation retention [32]. Likewise, it can be speculated that the absence of L-lysinylation of phospholipids in the plasma membrane of MprF-deficient mutants may also increase bivalent metal ion binding because of the increased negative charge of the cell surface, although this still needs to be demonstrated. However, loss of L-lysinylated phospholipids may also influence HBA resistance in a more complex and indirect way, since it was shown to induce profound changes in the lipidome and cellular physiology of *L. monocytogenes* and *E. faecalis* [50,79].

Because of their powerful activity, natural origin, and low toxicity, HBAs are of potential interest for various clinical and industrial applications [22,23,80]. The finding in the present work that HBA-resistant mutants display collateral sensitivity to cationic antimicrobials, such as nisin and polymyxin B, indicates that a combination or alternation of these compounds could increase their potency and robustness in such applications. As resistance to one compound implies sensitivity to the other, the emergence of resistance in the presence of both compounds may be limited. Similar to our findings, deletion of *mprF* in *L. monocytogenes* was already found to increase resistance to sodium orthovanadate, chloroxylenol, and niaproof [50], but HBAs are the first nontoxic, food-grade compound with such an effect. Notably, the same authors reported that deletion of the same gene in *B. subtilis* led to decreased resistance to chloroxylenol, which underscores that membrane lysinylation has complex species-dependent implications [50]. As HBAs have a remarkably strong antimicrobial potency, especially at low pH, more research on the antimicrobial potency of combinations of HBA with other compounds is warranted to provide additional insights into these mechanisms and to fully exploit their application potential based on the phenomenon of collateral sensitivity.

In conclusion, our research provides the first insights into a specific mechanism of resistance development of *L. monocytogenes* against HBA and, thereby, contributes to a better understanding of the antimicrobial mechanism of HBA. The finding that the elimination of either MprF or DltABCD increases the resistance to this hop compound suggests a role of the cell surface charge in the mechanism. Finally, we demonstrated that the evolution of HBA resistance via this mechanism results in collateral sensitivity to cationic antimicrobials, and this may strengthen the case for using HBA as a natural and nontoxic antimicrobial in various applications.

## Figures and Tables

**Table 1:** Strains and plasmids used in this work. The superscript “r” after an antibiotic denotes resistance.

Strain		Description	Reference
*L. monocytogenes*			
	WT	Wild-type strain Scott A, serotype 4b	[36]
	WT/pI	WT with pIMK2 integrated; Km^r^	This work
	HBA2.2	Isolated HBA-resistant mutant	This work
	HBA2.7	Isolated HBA-resistant mutant	This work
	*ΔarsB1*	*arsB1* deletion mutant	This work
	*ΔmprF*	*mprF* deletion mutant	This work
	*ΔmprF/*pI	*mprF* deletion mutant with integrated pIMK2; Km^r^	This work
	*ΔmprF/*pI*-mprF*	*mprF* deletion mutant with integrated pIMK2-*mprF*; Km^r^	This work
	*ΔdltA*	*dltA* deletion mutant	This work
	*ΔdltA/*pI	*dltA* deletion mutant with integrated pIMK2; Km^r^	This work
	*ΔdltA/*pI*-dltABCD*	*dltA* deletion mutant with integrated pIMK2-*dltABCD*; Km^r^	This work
	*ΔmprF/ΔdltA/*pI	*mprF* and *dltA* deletion mutant with pIMK2 integrated; Km^r^	This work
*E. coli*			
	DH5α	Cloning host strain	[37]
	S17-1λpir	Cloning host strain and donor strain for plasmid conjugation	[38]
**Plasmids**		**Description**	**Reference**
pIMK2		Site-specific integrative vector, constitutive overexpression promoter Phelp, 6.2 kb, Km^r^	[39]
pIMK2-*mprF*		Phelp-driven constitutive MprF overexpression	This work
pIMK2-*dltABCD*		Phelp-driven constitutive DltABCD overexpression	This work
pHoss1		Temperature-sensitive suicide plasmid for gene deletion, 9 kb, Amp^r^, Ery^r^	[40]
pHoss1-oriT		pHoss1 plasmid with integrated RP4 oriT from pIMK2, 9.2 kb, Amp^r^, Ery^r^	This work
pHoss1-oriT*ΔmprF*		pHoss1-oriT carrying an in-frame MprF deletion cassette	This work
pHoss1-oriT*ΔarsB1*		pHoss1-oriT carrying an in-frame ArsB1 deletion cassette	This work
pHoss1-oriT*ΔdltA*		pHoss1-oriT carrying an in-frame DltA deletion cassette	This work

**Table 2:** Oligonucleotide primers used in this work. Restriction sites for SalI and BamHI are underlined, overhangs for Gibson assembly are italicized. F and R indicate forward and reverse primer, respectively.

Primer	Oligonucleotide Sequence (5′-3′)
NC16(2)	GTCAAAACATACGCTCTTATCGATTC
pIMK_F	GGGTTTCACTCTCCTTCTAC
pIMK_R	GGTACCCAGCTTTTGTTCC
pHoss1_F	GCGTCGACGTCATATGGATC
pHoss1_R	CTCCCGGGTACCATGGGATC
mprF_SalI	TACTGTCGACCTATTTGCAAGTGGTC
mprF_BamHI	CTCGGGATCCATGAAAGAAAAATTAATGCAAGCC
mprF_A	*TAGATCCCATGGTACCCGGGAG*CTCCTTCTCCTATCTATCATACC
mprF_B	*ACTCTGTTATG*ACTTGCAAATAGTCAAGTAGTC
mprF_C	*TATTTGCAAGT*TTTCATAACAGAGTCTCCTTAAC
mprF_D	*CGGATCCATATGACGTCGACGC*GCTTTACCTAGATTTAGGATACTG
dltABCD_F	*GGGAACAAAAGCTGGGTACC*TTACTTACGGTCTTTTTTTG
dltABCD_R	*GTAGAAGGAGAGTGAAACCC*ATGACAACGAGTATCATAGAAAG
dltA_A	*GATCCCATGGTACCCGGGAG*GAAACAGGTACAGTGATATC
dltA_B	*CATAGAAAGA*GAGGTTAACAAGTGAGTTTAC
dltA_C	*TGTTAACCTC*TCTTTCTATGATACTCGTTG
dltA_D	*GATCCATATGACGTCGACGC*GTAACTGCTATTCCCTTC
arsB1_A	*TAGATCCCATGGTACCCGGGAG*TTATCTGGTTCTTGTACAGTAGAG
arsB1_B	*CTTCTTAACGA*CATCTTCCTCCATCATAAAATTCAAATAAG
arsB1_C	*GGAGGAAGATG*TCGTTAAGAAGCTAGTTTAAGAAG
arsB1_D	*CGGATCCATATGACGTCGACGC*CTAACTCTTCAACTTCATCTCC

**Table 3:** MIC values of *L. monocytogenes* WT and *mprF* and *dltA* mutants for additional cationic and anionic antimicrobials.

	Anionic			Cationic	
Strain	HBA (ppm)	Na Deoxycholate (µg/mL)	SDS (µg/mL)	PMB (µg/mL)	Nisin (µg/mL)
WT	2	625	250	100	75
*ΔmprF*	3	625	250	50	18.25
*ΔdltA*	2	625	250	50	37.5
*ΔmprF*/*ΔdltA*	3	625	250	12.5	9.125

## Data Availability

Data are contained within the article.
